# 伴KMT2A::MAML2融合基因急性淋巴细胞白血病转急性髓系白血病1例

**DOI:** 10.3760/cma.j.cn121090-20231203-00290

**Published:** 2024-02

**Authors:** 艳 张, 静波 倪, 琼洁 张, 爽 惠, 春芳 王, 彤 王

**Affiliations:** 河北燕达陆道培医院，廊坊 065201 Hebei Yandaludaopei Hospital, Langfang, 065201, China

女，16岁，2017年3月诊断急性B淋巴细胞白血病（B-ALL）；行化疗后（具体方案不详）达到缓解，至2019年12月24日停用6-巯基嘌呤、甲氨蝶呤，停药时骨髓为缓解状态。2020年3月2日血常规：WBC 3.87×10^9^/L，HGB 108 g/L，PLT 69×10^9^/L。2020年3月30日入我院，骨髓象：增生活跃，幼稚淋巴细胞占有核细胞1％。免疫分型：①未见恶性幼稚B淋巴细胞。②CD34^+^、CD117^+^髓系原始细胞占有核细胞0.25％。HLA-DR、CD33表达减低，部分异常表达CD7、CD96。融合基因筛查阴性（常规检测未包括KMT2A::MAML2融合基因）。白血病少见融合基因及融合基因家族成员分析：KMT2A::MAML2融合基因阳性，融合方式1为KMT2A基因的9号外显子与MAML2基因的3号外显子发生融合；融合方式2为KMT2A基因的10号外显子与MAML2基因的3号外显子发生融合。血液系统遗传病相关基因筛查：CFD、MCM4突变基因阳性。骨髓染色体核型：46,XX,del（6）（p23）,add（10）（p13）,inv（11）（q21q23.3）×2[27]，外周血染色体核型正常。骨髓FISH检测KMT2A双色断裂分离探针：KMT2A基因分离信号间期核占85％（单基因分离间期核35个，占7％，双基因同时分离间期核390个，占78％），提示染色体水平inv（11）（q21q23.3）累及KMT2A基因结构异常。初诊时骨髓涂片FISH检测KMT2A双色断裂分离探针：未见KMT2A基因重排。外周血先天骨髓衰竭综合征基因突变分析检查：阴性。骨髓活检：白血病治疗后，骨髓纤维化，幼稚前体细胞比例未见明显升高，巨核细胞增生较活跃伴发育异常。免疫组化：CD20（少量B淋巴细胞+），CD3（少量T淋巴细胞+），CD138（少量浆细胞+），CD117（少量+），CD163（散在+），CD34（个别+），MPO（粒系+），CD61（巨核细胞+），TdT（少量+），CD71（红系+）。患者未应用升白细胞药物等治疗，门诊监测血常规指标逐步恢复。2020年5月19日血常规：WBC 4.25×10^9^/L，HGB 115.8 g/L，PLT 404×10^9^/L，中性粒细胞2.3×10^9^/L。2020年5月25日复查骨髓免疫残留：未见恶性幼稚细胞。白血病融合基因筛查：阴性。染色体核型：46,XX,del（6）（p23）,add（10）（p13）,inv（11）（q21q23.3）×2[2]/46,XX[28]。FISH检测KMT2A双色断裂分离探针：KMT2A双基因分离间期核3个，占0.6％，异常比例较前显著降低。骨髓象：增生活跃，局部增生明显活跃，幼稚淋巴细胞1％。2020年7月19日复查血常规：WBC 3.18×10^9^/L，HGB 111 g/L，PLT 75×10^9^/L。骨髓象：增生活跃，原始粒细胞21％，诊断AML-M_2_。白血病融合基因筛查：KMT2A-PTD阳性。骨髓免疫分型：未见恶性幼稚B淋巴细胞，33.27％恶性幼稚髓细胞。血液肿瘤突变组分析（86种）：CBL A758D突变阳性。2020年7月28日FISH检测KMT2A双色断裂分离探针：KMT2A基因分离信号间期核占80％，单基因分离（1R1G1F）间期核占8％，双基因分离（2R2G）的间期核占72％。染色体核型：46,XX,del（6）（p23）,add（10）（p13）,inv（11）（q21q23.3）×2[8]/46,XX,del（6）（p23）,del（6）（q13q23）,add（10）（p13）,inv（11）（q21q23.3）×2[11]/46,XX[1]。予地西他滨20 mg/m^2^（第1～5天）+阿糖胞苷10 mg/m^2^每12 h 1次（第3～16天）+阿克拉霉素7 mg/m^2^（第4～11天）+重组人G-CSF 200 µg/m^2^（第3～16天）+维纳妥拉口服化疗治疗。过程中出现过敏反应暂停化疗，予抗过敏等治疗。病情好转继续化疗。2020年8月5日患者出现发热，考虑感染，予其加左氧氟沙星、头孢曲松、美罗培南抗感染治疗。血培养汇报：生长大肠埃希菌。2020年8月17日骨髓流式细胞术检测：未见恶性幼稚细胞。KMT2A-PTD基因定量阴性。2020年9月5日开始行供者为非血缘的异基因造血干细胞移植治疗，HLA分辨率10/10，血型A供O，预处理方案为Ara-c/Bu/Cy/ATG/Me-CCCNU，应用米芙+他克莫司+甲氨蝶呤预防GVHD。2020年9月17日回输供者外周干细胞，共回输单个核细胞4.72×10^8^/kg，CD34^+^细胞2.79×10^6^/kg。2020年9月18日回输第三方脐带血。+12 d白细胞植活，+9 d血小板植活。移植后定期复查骨髓，均为完全缓解状态。移植后出现急性GVHDⅢ度（肝脏3级，肺部），移植6个月后死亡。

